# The relationship between learning speed and personality is age- and task-dependent in red junglefowl

**DOI:** 10.1007/s00265-018-2579-2

**Published:** 2018-09-26

**Authors:** Josefina Zidar, Alexandra Balogh, Anna Favati, Per Jensen, Olof Leimar, Enrico Sorato, Hanne Løvlie

**Affiliations:** 1 Department of Physics, Chemistry and Biology, IFM Biology, Linköping University, 58183 Linköping, Sweden; 2 Department of Zoology, Stockholm University, 10691 Stockholm, Sweden

**Keywords:** Exploration, Cognition, *Gallus gallus*, Personality

## Abstract

**Abstract:**

Cognition is fundamental to animals’ lives and an important source of phenotypic variation. Nevertheless, research on individual variation in animal cognition is still limited. Further, although individual cognitive abilities have been suggested to be linked to personality (i.e., consistent behavioral differences among individuals), few studies have linked performance across multiple cognitive tasks to personality traits. Thus, the interplays between cognition and personality are still unclear. We therefore investigated the relationships between an important aspect of cognition, learning, and personality, by exposing young and adult red junglefowl (*Gallus gallus*) to multiple learning tasks (discriminative, reversal, and spatial learning) and personality assays (novel arena, novel object, and tonic immobility). Learning speed was not correlated across learning tasks, and learning speed in discrimination and spatial learning tasks did not co-vary with personality. However, learning speed in reversal tasks was associated with individual variation in exploration, and in an age-dependent manner. More explorative chicks learned the reversal task faster than less explorative ones, while the opposite association was found for adult females (learning speed could not be assayed in adult males). In the same reversal tasks, we also observed a sex difference in learning speed of chicks, with females learning faster than males. Our results suggest that the relationship between cognition and personality is complex, as shown by its task- and age-dependence, and encourage further investigation of the causality and dynamics of this relationship.

**Significance statement:**

In the ancestor of today’s chickens, the red junglefowl, we explored how personality and cognition relate by exposing both chicks and adults to several learning tasks and personality assays. Our birds differed in personality and learning speed, while fast learners in one task did not necessarily learn fast in another (i.e., there were no overall “smarter” birds). Exploration correlated with learning speed in the more complex task of reversal learning: faster exploring chicks, but slower exploring adult females, learned faster, compared to less explorative birds. Other aspects of cognition and personality did not correlate. Our results suggest that cognition and personality are related, and that the relationship can differ depending on task and age of the animal.

**Electronic supplementary material:**

The online version of this article (10.1007/s00265-018-2579-2) contains supplementary material, which is available to authorized users.

## Introduction

Cognition defines how individuals perceive, process, store, and act on environmental stimuli, and includes perception, learning, and decision-making (Dukas 2004; Shettleworth 2010). Cognitive processes influence behavior that can have fitness consequences (for review see Dukas 2004; Shettleworth 2010). Yet, individual variation in cognition has been relatively understudied in non-human animals (Sih and Del Giudice 2012; Thornton and Dukas 2012; Griffin et al. 2015; Shaw and Schmelz 2017; Boogert et al. 2018). Among the potential factors accounting for between-individual differences in cognitive traits (e.g., learning, memory) are sex differences (e.g., Range et al. 2006; Halpern 2012) and genetic variation (see Dukas 2004 for review). For example, male ravens (*Corvus corax*) learned an association faster than females (Range et al. 2006), and selection on learning performance in fruit flies (*Drosophila melanogaster*) led to a dramatic divergence in learning performance between two selection lines (Lofdahl et al. 1992). Animal personality (i.e., consistent differences among individuals in behavioral responses, aka coping styles or behavioral types; Gosling 2001; Dall et al. 2004; Sih et al. 2004) has been hypothesized to be functionally related to individual differences in cognition (Sih and Del Giudice 2012; Griffin et al. 2015). Among studies that have investigated this relationship in non-human animals, most have focused on learning speed and have related this trait to personality traits (recently reviewed by Dougherty and Guilliette 2018) such as boldness (e.g., guppies, *Poecilia reticulata*, Dugatkin and Alfieri 2003; Eastern water skink, *Eulamprus quoyii*, Carazo et al. 2014), activity (e.g., mice, *Mus musculus*, Matzel et al. 2006; zebra finches, *Taeniopygia guttata*, Brust et al. 2013), exploration (e.g., great tits, *Parus major*, Titulaer et al. 2012; black-capped chickadees, *Poecile atricapillus*, Guilette et al. 2009, 2011, 2015; mallards, *Anas platyrhynchos*, Bousquet et al. 2015), and neophobia (Florida scrub-jays, *Aphelocoma coerulescens*, Bebus et al. 2016).

In contrast to the relatively limited number of studies on this in animals, in human personality research, there is a long tradition of relating personality to cognitive styles (i.e., the way individuals acquire, process, store, and act on information, independent of cognitive ability, e.g., Eysenck 1978, 1981; Furnham 1992; Sih and Del Giudice 2012). For example, extroverts responded quickly to information as opposed to more reflective introverts (Furnham 1992). However, cognition and personality are complex constructs and their interrelationship is still debated and not fully resolved (Griffin et al. 2015; Volkova and Rusalov 2016, and references therein).

In animals, the main framework linking variation in personality and cognition is centered on a speed–accuracy trade-off (Sih and Del Giudice 2012), which predicts that individuals may employ different cognitive styles (being either fast or accurate) based on personality or coping style. Proactive individuals (typically described as bold, aggressive, fast explorers, Benus et al. 1990; Koolhaas et al. 1999, 2010; Coppens et al. 2010) are expected to adapt a speed-over-accuracy strategy and learn simple tasks quickly, but make more mistakes when tasks become more difficult (Sih and Del Giudice 2012). Reactive individuals, which adopt a strategy of accuracy over speed, are instead predicted to learn new tasks relatively slowly compared to proactive individuals, but to pay more attention to changes, and therefore make fewer errors when tasks require responding to a change of environmental stimuli. Individuals are thus expected to differ in learning speed depending on personality, but the relationship is also expected to be task-specific (i.e., varying with the presented task, Koolhaas et al. 2010; Sih and Del Giudice 2012; Griffin et al. 2015). However, empirical research exploring speed–accuracy trade-offs report somewhat unclear results. For example, in some species, reactive individuals performed better at both avoidance learning (Exnerová et al. 2010) and reversal learning tasks (black-capped chickadee, Guillette et al. 2011; cavy, *Cavia aperea*, Guenther et al. 2014), while in others, there were no differences (Amy et al. 2012; Guillette et al. 2015). Yet, other studies found a nonlinear relationship between spatial learning and boldness (Eastern water skink, Carazo et al. 2014). Taken together, these findings support that the relationship between learning speed and personality can be complex, and possibly species-dependent, and imply that a speed–accuracy trade-off may not always underlie observed relationships between cognition and personality.

A limitation of recent studies is that they typically assess cognitive performance in a single task and relate this to a single personality trait (Griffin et al. 2015). Here we have included multiple measures for both cognition and personality. However, to improve our understanding of the nature and generality of the observed relationship between personality and cognition, performance across different cognitive domains should be more broadly assayed (Griffin et al. 2015).

In order to shed light on these issues, we investigated the relationship between variation in learning across several commonly used cognitive tasks, ranging from relatively simple ones to more complex tasks, and commonly used measures of personality in fowl, in both juvenile and adult red junglefowl (*Gallus gallus*) (Favati et al. 2016; Zidar et al. 2017a, b). In other bird species, some studies report sex differences in learning where males are faster than females (e.g., great tits, Titulaer et al. 2012; zebra finches, Brust et al. 2013), while other studies find females to be faster than males (e.g., great tits, Brodin and Urhan 2015). A recent meta-analysis confirms that there can be large differences between the sexes (Dougherty and Guillette 2018). Whether learning speed across tasks correlate or not also differs among avian species (e.g., correlate: North Island robins, *Petroica longipes*, Shaw et al. 2015, Australian magpies, *Cracticus tibicen dorsalis*, Ashton et al. 2018; correlate negatively: Florida scrub-jays, Bebus et al. 2016; do not correlate: black-capped chickadees, Guilliette et al. 2015; song sparrows, *Melospiza melodia*, Andersson et al. 2017). Regarding the correlation between personality and learning specifically, in great tits selected for fast and slow exploration, one study found that exploration did not explain variation in discriminative learning, but that birds from the slow selection line took more trials to learn a reversal learning task (Amy et al. 2012), while another study showed that slow female explorers outperformed fast explorers in discrimination learning (Titulaer et al. 2012). In zebra finches, more active and fearful individuals learned faster (Burst et al. 2013); in mallards, fast-exploring individuals took longer to accomplish a spatial task (Bousquet et al. 2015), while in black-capped chickadees, exploration did not explain variation in learning speed (Guilliette et al. 2015). A recent meta-analysis that explored the link between personality traits (exploration, boldness, activity, aggression, and sociability) and cognition (initial learning/reversal speed, number of correct choices/errors after standard training) found a significant association between variation in personality and variation in cognition (Dougherty and Guillette 2018). The direction of these relationships was vastly variable (Dougherty and Guillette 2018). Based on this, our study was set out to explore (i) sex differences in learning speed, (ii) learning speed across tasks, and (iii) how learning speed is linked to personality, in chick and adult red junglefowl. Based on previous studies, we predict that males and females will differ in learning speed (Titulaer et al. 2012; Brust et al. 2013; Brodin and Urhan 2015), learning speed co-varies across tasks, showing a positive correlation (e.g., Ashton et al. 2018), that there will be an association between personality and cognition (Dougherty and Guillette 2018) where fast explorers learn faster in simpler tasks (i.e., our discriminative and spatial learning task), while slow explorers learn faster in more complex tasks (i.e., our reversal learning task, Sih and Del Giudice 2012).

## Methods

### Animals and housing

We conducted personality and cognitive assays between March and September 2013 on a captive population of red junglefowl reared at Linköping University, Sweden. All birds originated from two sources (Copenhagen Zoo and Götala Research station, Skara) held in captivity for over 12 generations (Schütz and Jensen 2001) and pedigree bred since 2011. Founders and study populations have been randomly bred and not subject to any intentional selection, and our study animals look and behave similarly to their wild ancestors (Schütz and Jensen 2001). All individuals were artificially incubated and reared together without exposure to their mothers, thereby reducing any influence that maternal effects and accumulated ontogenetic experiences may have on development of cognitive abilities and personality (Stamps and Groothuis 2010).

To be able to assay a large number of individuals across different cognitive tasks and at the same age, we used two batches of chicks, separated by 3 weeks, totaling 100 individuals (*n*_females_ = 55, *n*_males_ = 45, *n*_families_ = 18). All chicks were marked with wing tags to facilitate recognition and housed in same-age, mixed-sex groups (1–3 groups per batch) in indoor enclosures (0.5–3 m^2^, size increasing with the age of the chicks). Not all chicks took part in all cognitive tasks (see below), and test individuals were chosen to represent all available families. Eight weeks post-hatching, following the first set of cognitive tasks and personality assays (see below), 87 individuals (*n*_females_ = 45, *n*_males_ = 42) were moved to an experimental chicken facility, at Linköping University, and kept in a single group until sexual maturity when birds were divided into two groups according to sex*.* All birds had ad libitum access to water, commercial poultry food, dust bath, and perches, but were deprived of mealworms (used as reward) between test trials.

### Experimental setup

In all tests, individuals were tested singly, and between 8 and 18 local time (lights were on 7–19), following descriptions of previous testing in the same population (e.g., Zidar et al. 2017a, b; Sorato et al. 2018). Sex of subjects (because young chicks are monomorphic) and their personality (since personality was measured after cognitive testing) were not known to observers at the time of cognitive testing; thus, we aimed to minimize observer bias.

#### Cognitive testing of chicks

##### Discrimination learning

Chicks were handled from day 1 post-hatching and gently habituated to the test arena (28 × 18 × 37 cm, see SI, Fig. S1), and to temporary isolation from their pen mates. Three days post-hatching, the ability to learn a simple association was tested (*n*_females_ = 35, *n*_males_ = 27). Chicks had to associate a conditioned stimulus (CS), randomly assigned to be blue or green color, with an unconditioned stimulus (US), ca. one third of a mealworm (see Zidar et al. 2017b, for detailed information on the test setup). Vision is the primary sense in many bird species including the fowl, and novel colors are readily learned in chickens (Osorio et al. 1999; Zylinski and Osorio 2013). We measured reflectance of colors with a light spectrometer (Ocean Optics, Dunedin, FL, USA, USB2000 PX-2 pulsed xenon light source spectrometer), confirming that they are perceived as separate colors by domestic fowl, with the same intensities and color distances from the achromatic point in a two-dimensional color space describing photoreceptor stimulations in the chick eye (see Osorio et al. 1999). Chicks were allowed to make a discriminative choice by moving from a set starting point at one end of the arena to the other end where two bowls (one green and one blue) were placed (ca. 20 cm away, see Fig. S1). The reward in the bowl could not be seen by the chick until just above the bowl. The chick was placed with its head facing away from the bowls and a trial started as soon as the chick turned around and was facing the bowls (i.e., a “trial” was defined as when a chick walked from the starting position to the bowls). The trial ended as soon as the chick had made its choice and eaten a mealworm (i.e., even if a chick chose the incorrect bowl, it was allowed to inspect the correct bowl and consume the mealworm). To reduce individual variation in learning performance due to different levels of habituation or emotional state (i.e., fear, anxiety), each chick was allowed the amount of time it needed to make a choice (which usually occurred within seconds).

To decrease the possibility that a chick would make unilateral choices, i.e., that it may associate the reward with its position rather than the color cue, the stimuli changed place (left–right) between each trial. An additional side preference test showed no side bias at this age (days 6 and 17, see Fig. S2). After each trial and while the two cues were repositioned, the chick was held outside the arena, precluding the possibility to observe the positioning of the reward. A session lasted for a maximum of 15 min; the session could be shorter depending if a chick was not motivated in the test. Each chick was allowed a maximum of eight training sessions, with minimum 1 h of rest between sessions, over 2 days to learn the discriminative task. This was enough for all except one individual to learn the tasks. We assumed that a chick had learned the task if it met a criterion of five correct choices in a row in a single session. If a chick had learnt the task late in the afternoon, it was not tested any further in the day, but instead went through a “refresh trial” in the following morning (which was not included in our measure of trials needed to reach our learning criterion because it took place after our criterion was reached), where it again had to make five correct choices in a row before continuing to the next learning task. This was done to ensure that the association between the stimuli and reward was salient before moving on to reversal learning (see below). In simulations under the null hypothesis scenario of random choice, only an average of 3% of our putative learners could have passed this criterion by chance (Sorato et al. 2018). The total number of trials (choices) until the criterion was reached provided a measure of individual “learning speed.”

##### Reversal learning

After reaching the criterion for discrimination learning, chicks were assayed in a reversal learning task: the association between cue and reward was reversed, so that the previously unrewarded stimulus was now rewarded and vice versa. The reversal learning task was conducted in a similar fashion as the discrimination learning task with regard to the amount of time given, number of training sessions (with up to eight training sessions over 4 days), alteration of the side of stimuli, measure of learning speed, and learning criterion (Zidar et al. 2017b). Four birds did not learn to associate the reversed stimulus with a reward within the given time frame and were therefore not included in the analysis (sample size analyzed: *n*_females_ = 33, *n*_males_ = 24).

##### Spatial learning

At 5 weeks of age, the previously tested chicks (*n* = 62) were trained to learn the location of a food reward (a mealworm in a bowl) located 0.5 m after a turn in a U-shaped arena (76 × 114 cm, Fig. S3, Zidar et al. 2017a, b). The chicks could not see the reward from the starting position. This has similarities to spatial tasks used in rodents (Benus et al. 1990), and, although simple, we call it a spatial task. Chicks were trained to form a routine and move directly to the reward after release. If the task was performed five times in a row within the same session, without the chick stopping or turning around (Zidar et al. 2017b), the chick was assumed to have learnt. The number of trials needed to reach this criterion was used as “learning speed.” Six individuals did not engage in the task and were not able to learn the task and were therefore not included in the analysis (sample size analyzed: *n*_females_ = 30, *n*_males_ = 26).

For all chicks, each training session lasted ca. 15 min, unless a chick lost motivation for the reward (e.g., did not want to eat the mealworm, were distress calling, or tried to fly out of the test arena). If a training session ended before a routine had been formed, a new session commenced after approximately 1 h of rest.

#### Cognitive testing of adult female fowl

##### Discrimination learning

While adult females readily work for food rewards, sexually mature male fowl (≥ 5 months of age, Johansen and Zuk 1998) are harder to motivate to engage in a task using food as reward. Re-testing of learning speed therefore only included females (*n* = 45). To increase the sample size, we included both females that had been tested in the cognitive tasks as chicks (*n* = 27) and females (*n* = 18) that were not. Adult females were tested in a lab room adjacent to their home pen. To avoid memory effects, we used two novel cues consisting of two different color patterns (white background with black circle or black background with white circle; the amounts of black and white were 50–50%). Both novel colors and contrast patterns are readily learned in chickens (Osorio et al. 1999; Zylinski and Osorio 2013); thus, we did not expect differences in task difficulty for chicks and adults. The discriminative task for adults differed somewhat from that used in chicks and was adjusted because of the difference in behavior between chicks and adults. Females were trained to associate one of the two patterns with a reward (mealworm), hidden in a bowl underneath a patterned lid. Same as for chicks, females were randomly assigned to be trained on either of the two stimuli, while the left–right position of the two patterns now changed between trials in a random manner (role of a dice). Training took place on a table with females facing the tester and the two bowls. Three steps were used to facilitate learning: (i) reward was presented on top of the lid, (ii) reward was placed in the bowl half-covered by the lid so that the fowl could see the worm, and finally (iii) the bird had to remove the lid to reach the reward under it. Females were trained to remove the lid to obtain the reward and a choice was noted as “correct” if the rewarded stimulus was chosen. Females were only allowed one choice per trial and were not allowed to collect the reward if they chose the incorrect stimulus. A black shield was placed in front of the female so that she could not see when the stimuli changed places. Similarly, as earlier described for chicks, the criterion for learning was five correct choices in a row, and the number of trials needed until this was obtained was recorded as “learning speed.” All three steps of learning were included when measuring learning speed. All individuals got exactly 100 trials over six training sessions over 2 days to learn the association, enabling us to estimate learning curves (see Fig. S4a). Consistent with learning, individuals chose at random at the beginning of the discrimination test, and the proportion of correct choices increased steadily over trials towards 80% of correct choices (calculated in learning blocks of 5, Fig. S4a). Eleven females failed to learn the association and were not included in the analysis (where 7 were previously tested as chicks).

##### Reversal learning

Following the discrimination learning task, females that had succeeded in associating a stimulus with a reward (*n* = 34) were exposed to a reversal learning task. No pre-training was required and the task was conducted in the same way as for the discrimination learning task described above and for 100 trials, except that now the previously unrewarded stimulus was rewarded while the previously rewarded stimulus was not. Females showed a high proportion of errors (80%) at the beginning of the test (matching the rate at the end of the previous discriminative test), and rate of correct choices increased with test progression and reached a stable level of about 80% correct choices (calculated in learning blocks of 5) at the end of the test (Fig. S4b). Twenty-seven females successfully learned the task, while 7 females failed to learn the task and were excluded from the analysis (where 6 of the latter were previously tested as chicks).

#### Personality assays for chicks

All individuals were tested in three personality assays (a novel arena, novel object, and tonic immobility test, see below), at 4 and 6 weeks of age (Zidar et al. 2017a, b).

##### Exposure to a novel arena

To quantify variation in exploration and boldness, a novel arena test was performed (Forkman et al. 2007; Réale et al. 2007). Empty, familiar food, and water containers were placed in the arena (76 × 114 cm) to obscure the chicks’ full view and encourage exploration. The substrate was changed (first test occasion: wood shavings; second test occasion: shredded cardboard paper) and placement of containers altered to keep the environment novel between the test occasions (Fig. S6a, b). A chick was gently caught from its home pen and placed at one of the arena’s short sides during darkness. Behavioral measures of its responses started when the lights were turned on again. To prevent the chicks from escaping, a metal grid was placed over the arena. To be able to observe if the birds used the entire arena or only parts of the arena, we divided the arena into six equally sized (imagined) sections (i.e., the birds could not see these sections) and scored how many of these imaginary sections the bird visited during the test. Behaviors were scored live via video cameras connected to computer screens using an instantaneous recording rule every 10 s for the 10 min the test lasted. Latencies were on the other hand scored continuously (i.e., as exact latencies) and behaviors that occurred in very low frequencies were recorded continuously as number of occurring events (Table 1). Behaviors were based on previous personality studies in the fowl (Favati et al. 2014, b, 2016; Zidar et al. 2017a, b). Each chick was scored by one observer out of a total of four, and scoring was blind with regard to the bird’s scores on previous cognitive tests.

**Table 1:** Behaviors recorded in the novel arena and novel object test

Behavior	Description
Latency to move	Latency until the bird started moving
Latency to visit all areas of the arena	Latency until the bird had visited all 6 imagined sections of the arena
Locomotion	Frequency of locomotion; walking, running, and not foraging
Foraging	Frequency of time spent with its head down close to the ground either pecking at the floor or scratching the surface with its feet
Vigilance	Frequency of time spent standing or walking with its eyes open and head high above shoulder-height
Number of escape attempts	Total number of times the chick tried to leave the arena, e.g., by trying to jump or fly out

All latencies are recorded in seconds from when the bird first was placed in the test arena

##### Exposure to a novel object

To score variation in boldness and neophobia (Réale et al. 2007), a novel object (a spherical, brown/beige plush toy measuring 15 cm and with ca. 2-cm large yellow and black eyes) was placed in the arena. The novel object was placed along one of the short sides inside the arena directly after the novel arena test had finished. This occurred after birds had had 10 min to familiarize themselves with the test arena, which reduce confounding effects of a novel object being presented in a novel environment (Réale et al. 2007). Because the toy may resemble a potential predator, we aimed to capture both neophobia and boldness (Greggor et al. 2015). Lights were turned off while placing the novel object in the arena (birds were standing still in darkness), as far away from the chick as possible. The same behaviors as described above were scored. The novel object test lasted for 10 min starting when the object was introduced and the lights were turned on again.

##### Tonic immobility

Tonic immobility is a commonly used test of fear responses in birds (Forkman et al. 2007). Birds were calmly collected from their home pen and tested in a lab room adjacent to the home pen. To induce tonic immobility, a chick was placed on its back in a V-shaped wooden stand (20 × 10 cm). A light pressure was applied to its breast while also loosely holding a hand over its head for 15 s. Thereafter, the pressure was released and latency until the chick moved its head (“Latency to move TI”) was recorded. If the chick jumped up on its feet within 3 s following the removal of the pressure by the hand, the procedure was repeated a total of three times. If the bird was still not induced into tonic immobility after three attempts, the chick received a score of 0 s. If the chick stayed immobile more than 10 min, it received a maximum score of 600 s and the test was terminated. The observer avoided direct eye contact with the chick, and the same observer did all tonic immobility tests. The observer was blind to the birds’ learning scores in other tests. The tonic immobility test was performed after the novel arena and novel object tests, and on a separate day from these tests, with the aim that birds had similar initial stress level prior to the test.

#### Personality assays for adult fowl

At 5 months of age, following sexual maturity, all individuals were again tested in novel arena, novel object, and tonic immobility tests. Novel arena and novel object tests followed each other, as previously described. The arena (2 × 2 m) had peat as substrate and as for chicks, empty, familiar food and water containers were placed within to encourage exploration. To reduce the risk of between-individual differences in stress level prior to the test, the birds were collected from their home pen in a calm manner and with the light switched off. The novel object had the same shape and size as used for chicks, but a different color (black/gray/white). The tonic immobility test was performed on a separate day in a lab room adjacent to the home pen. Behaviors were scored by direct observations. All three tests were otherwise performed and scored in the same manner as described above for chicks (Zidar et al. 2017b).

### Statistical analyses

All analyses and model selections described below were conducted in R version 3.2.2.

#### Individual consistency of responses

Rank order consistency in learning speed across cognitive and behavioral tasks within the same age class, and over time, were explored by the Spearman rank correlations. We adjusted *p* values for multiple comparisons using the false discovery rate procedure for multiple testing (Benjamini and Hochberg 1995).

#### Behavioral responses in personality assays

Some behaviors occurred in very low frequencies or showed little variation between individuals (freezing, standing, preening, laying down, foraging, other behaviors (e.g., flapping wings, shaking body)) and were not analyzed further. For the remaining behavioral variables (novel arena: “Locomotion,” “Vigilance,” “Latency to move,” “Latency to explore all areas”; novel object: “Vigilance” and “Number of escape attempts”, Table 1), we used the mean of behavioral responses obtained at 4 and 6 weeks, if correlations of responses obtained at 4 and 6 weeks showed the same direction for males and females. This resulted in pooled data for all behavior, except for “Latency to move TI” (Table S1). Accordingly, this variable was analyzed separately using only data from the behavioral responses obtained at 4 weeks of age (because this was obtained closer in time to cognitive testing).

Behavioral variables from novel arena and novel object tests were reduced by the use of a principal component analysis (PCA) with the R-package “FactoMineR.” Two distinct principal components with eigenvalues ˃ 1 were obtained for chicks (Table 2, Fig. S7) and adults (Table 2, Fig. S8), interpreted as describing individuals that are more or less exploratory (PC1, see Table 2) and shy (PC2, see Table 2).

**Table 2:** Principal component analysis of behavioral responses of red junglefowl chicks and adults in personality assays (novel arena, NA, and novel object, NO). The first principle component (PC1) is interpreted as primarily describing more or less exploratory individuals; the second (PC2) as mainly describing more or less shy individuals

	Chicks		Adults	
	PC1	PC2	PC1	PC2
Locomotion (NA)	*0.51*	− 0.20	*0.52*	*− 0.42*
Vigilance (NA)	*0.49*	0.06	*0.55*	0.20
Latency to move (NA)	*− 0.38*	0.28	0.04	*0.55*
Latency to explore all areas (NA)	*− 0.47*	*0.32*	*− 0.32*	*0.52*
Vigilance (NO)	*0.31*	*0.62*	*0.37*	*0.34*
Number of escape attempts (NO)	0.21	*0.63*	*0.43*	*0.30*
Eigenvalues	2.74	1.27	2.00	1.56
Variance explained (%)	45.67	21.13	33.39	26.07

Factor loadings, eigenvalues, and variance explained by components are presented. Values in italics have values > ± 0.30

#### Relationship between learning speed and personality

Across learning tasks, some birds failed to learn the task (between 1 and 11 individuals, dependent on task). Several of these birds failed to learn because they did not engage at all in the task, and as a consequence never had the possibility to learn the task. There are also potential problems with arbitrarily given maximum values (e.g., “ceiling effects,” Stamps and Groothuis 2010); therefore, we only analyzed learning speed of individuals that learned the tasks.

To investigate if there were any biases in learning that were due to the type of cue associated with the reward, we performed the Mann–Whitney *U* tests to assess whether learning speed differed according to the color (chicks) or pattern (adult females) that was matched to the reward.

To explore whether learning speed in the various tasks related to personality, we first evaluated normality by visual inspection of histograms of model residuals. To obtain homogenous variances on non-normally distributed count data (learning speed in discriminative learning and spatial learning), we used the function Box-Cox to find suitable transformations for our response variables. This resulted in log transformation of learning speed in the discriminative learning tasks for both chicks and adult females, and in the spatial learning task. Learning speed in the reversal learning task was approximately normally distributed for both chicks and adult hens and was therefore left untransformed. For chicks, models had Gaussian distribution with identity link and contained the variables “Sex” (i.e., male or female), “Exploration” (PC1), “Shyness” (PC2), “Sex*Exploration” (PC1), “Sex*Shyness” (PC2) as fixed effects, and “Family” added as a random effect. For adults, models did not include “Sex” because no adult males were tested. Models investigating the effect of variation in tonic immobility included the variables “Sex” (i.e., male or female), “Latency to move TI,” and “Sex*Latency to move TI” with “Family” as random effect. We explored full models containing all predictor variables. We centered factors so that zeros would correspond to the average value of the predictor. Results obtained following this approach were robust, because analyzing the data with other distributions (negative binomial or Poisson distribution) gave statistically similar results.

## Data accessibility

The datasets generated during and/or analyzed during the current study are available in Table S2 as a supplementary table.

## Results

### Individual consistency of responses

Based on individual rank order, learning speed was not correlated across learning tasks in either chicks or adult hens (Table 3, Fig. S5). Individual rank order of learning speed was not correlated in females over time (chick to adult) for either discrimination learning (*r*_s_ = − 0.12, *n* = 19, *p* = 0.62) or reversal learning (*r*_s_ = − 0.37, *n* = 12, *p* = 0.23).

**Table 3:** Spearman’s rank correlation among learning tasks: “Discrimination learning” (i.e., learning speed in the discrimination learning task), “Reversal learning” (i.e., learning speed in the reversal learning task), and “Spatial learning” (i.e., learning speed in the spatial learning task) for red junglefowl chicks (“♂♂” = males, “♀♀” = females, “♀♂” = both sexes) and adult females (“adult ♀♀”). None of the correlations were significant

	Discrimination learning	Reversal learning
	♂♂/♀♀/♀♂/adult ♀♀	♂♂/♀♀/♀♂/adult ♀♀
Reversal learning	*r* = − 0.27, *n* = 24/*r* = 0.07, *n* = 33/*r* = − 0.04, *n* = 57/*r* = 0.09, *n* = 27	–
Spatial learning	*r* = 0.14, *n* = 25/*r* = − 0.15, *n* = 30/*r* = − 0.03, *n* = 55/–	*r* = − 0.34, *n* = 23/*r* = 0.13, *n* = 29/*r* = − 0.14, *n* = 52/–

For behavioral responses in personality assays, individual consistency was present in chicks, between 4 and 6 weeks of age (*r*_s_ = 0.2–0.5, *n* = 100, *p* = 0.001–0.10, Table S1), but was weaker when comparing chicks and adults (*r*_s_ = 0.1–0.3, *n* = 87, *p* = 0.03–0.93, Table S1).

#### Learning speed of chicks

##### Discrimination learning

Individuals varied in number of trials needed to discriminate between the two colors (range, 7–64; mean ± SE, 24.02 ± 1.61), but learning speed was not explained by sex, personality, or their interaction (Sex: *χ*^2^ = 0.05, *df* = 1, *p* = 0.82; Sex effect from model with TI: *χ*^2^ = 0.02, *df* = 1, *p* = 0.89; Exploration: *χ*^2^ = 0.00, *df* = 1, *p* = 1.00; Shyness: *χ*^2^ = 0.05, *df* = 1, *p* = 0.82; TI: *χ*^2^ = 0.22, *df* = 1, *p* = 0.64; Sex*Exploration: *χ*^2^ = 1.05, *df* = 1, *p* = 0.30; Sex*Shyness: *χ*^2^ = 2.00, *df* = 1, *p* = 0.16; Sex*TI: *χ*^2^ = 0.86, *df* = 1, *p* = 0.35). Individuals found it easier to associate a blue color with a reward, than a green color (Mann–Whitney *U* test: *W* = 282.5, *p* = 0.009), but analyzing colors separately did not significantly change the results (blue: Sex: *χ*^2^ = 0.02, *df* = 1, *p* = 0.88; Exploration: *χ*^2^ = 0.26, *df* = 1, *p* = 0.61; Shyness: *χ*^2^ = 0.45, *df* = 1, *p* = 0.50; Sex*Exploration: *χ*^2^ = 0.48, *df* = 1, *p* = 0.49; Sex*Shyness: *χ*^2^ = 1.64, *df* = 1, *p* = 0.20; green: Sex: *χ*^2^ = 0.17, *df* = 1, *p* = 0.68; Exploration: *χ*^2^ = 0.24, *df* = 1, *p* = 0.63; Shyness: *χ*^2^ = 0.91, *df* = 1, *p* = 0.33; Sex*Exploration: *χ*^2^ = 0.05, *df* = 1, *p* = 0.82; Sex*Shyness: *χ*^2^ = 1.61, *df* = 1, *p* = 0.20).

##### Reversal learning

**Fig. 1:**
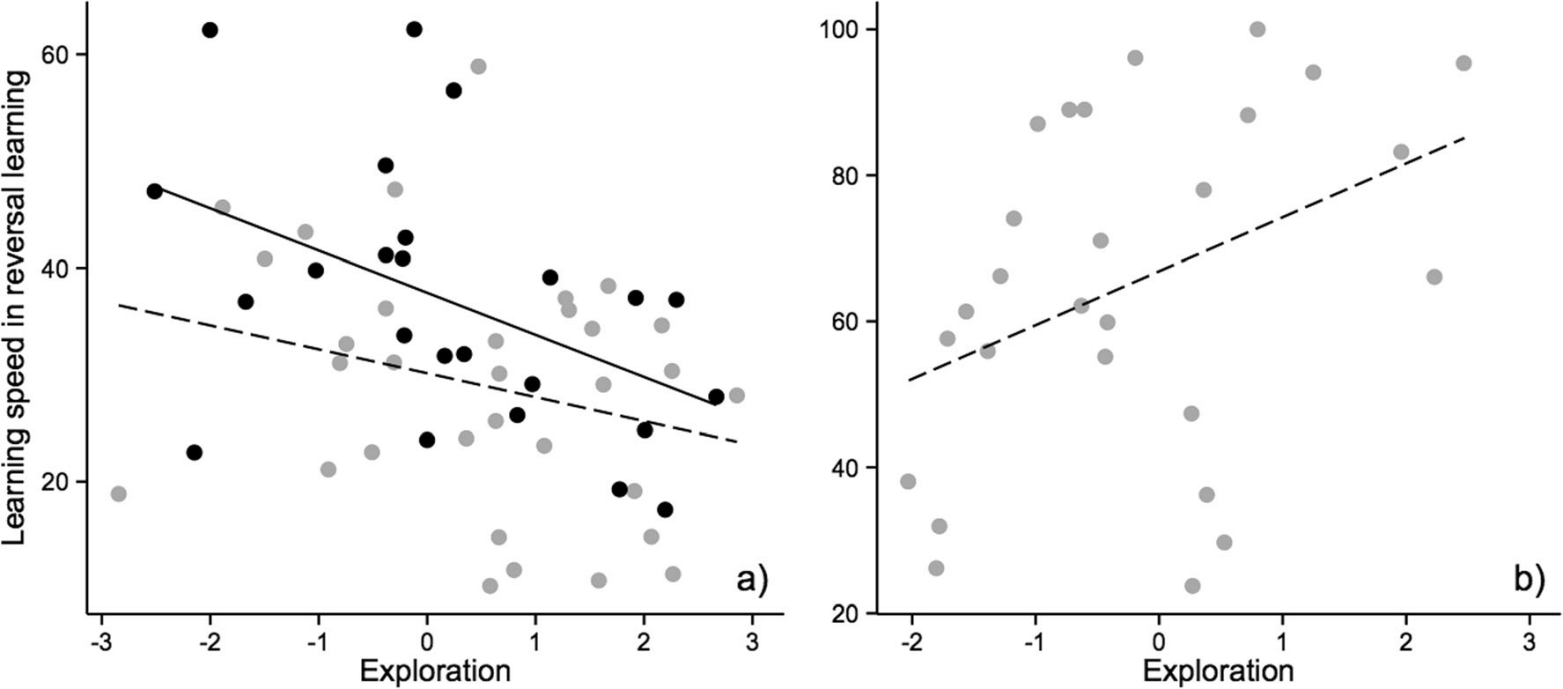
The relationship between learning (i.e., number of trials needed to reach the learning criteria) in a reversal learning task and exploration (PC1 from a PCA analysis) was **a** negative for chicks; more explorative individuals learned faster, and females (gray circles, dashed line) learned faster than males (black circles, solid line), and **b** positive for adult females where less explorative females learned faster. A single female outlier was removed from the sample (see the “Results” section for statistics including this data point)

Learning speed in the reversal learning task (range, 10–62; mean ± SE, 32.98 ± 1.76) was explained by both personality and sex. More explorative individuals (PC1) of both sexes learned the reversal task in fewer trials, compared to less explorative individuals (Exploration: *χ*^2^ = 4.67, *df* = 1, *p* = 0.03, estimate = − 1.91, Fig. 1a), and females learned faster than males (Sex: *χ*^2^ = 6.68, *df* = 1, *p* = 0.01, estimate = 8.56; Sex effect from model with TI: *χ*^2^ = 6.17, *df* = 1, *p* = 0.01, estimate = 7.75, Fig. 1a). Neither shyness, tonic immobility, nor interactions between personality variables and sex explained learning speed (Shyness: *χ*^2^ = 0.16, *df* = 1, *p* = 0.69; TI: *χ*^2^ = 0.00, *df* = 1, *p* = 0.95; Sex*Exploration: *χ*^2^ = 1.39, *df* = 1, *p* = 0.24; Sex*Shyness: *χ*^2^ = 0.43, *df* = 1, *p* = 0.51; Sex*TI: *χ*^2^ = 0.24, *df* = 1, *p* = 0.62). One female chick obtained an extreme exploration score, but analyses without this bird did not change the results qualitatively or quantitatively (Exploration: *χ*2 = 6.06, *df* = 1, *p* = 0.01; Shyness: *χ*^2^ = 0.02, *df* = 1, *p* = 0.89; Sex: *χ*^2^ = 4.56, *df* = 1, *p* = 0.03; Sex*Exploration: *χ*^2^ = 0.49, *df* = 1, *p* = 0.48; Sex*Shyness: *χ*^2^ = 0.15, *df* = 1, *p* = 0.70). There was no difference in the learning speed for blue and green colors in the reversal learning task (Mann–Whitney *U* test: *W* = 489.5, *p* = 0.12).

##### Spatial learning

Individual differences in spatial learning (range, 13–75; mean ± SE, 32.4 ± 2.05) were not explained by sex, personality, or their interaction (Sex: *χ*^2^ = 2.13, *df* = 1, *p* = 0.14; Sex in the model with TI: *χ*^2^ = 0.85, *df* = 1, *p* = 0.36; Exploration: *χ*^2^ = 0.60, *df* = 1, *p* = 0.40; Shyness: *χ*^2^ = 3.25, *df* = 1, *p* = 0.07; TI: *χ*^2^ = 0.86, *df* = 1, *p* = 0.35; Sex*Exploration: *χ*^2^ = 0.37, *df* = 1, *p* = 0.55; Sex*Shyness: *χ*^2^ = 0.10, *df* = 1, *p* = 0.75; Sex*TI: *χ*^2^ = 0.08, *df* = 1, *p* = 0.77).

#### Learning speed of adult females

##### Discrimination learning

Learning speed of adult females in the discrimination learning task (range, 15–93; mean ± SE, 46.32 ± 3.75) was not explained by personality differences (Exploration: *χ*^2^ = 0.01, *df* = 1, *p* = 0.93; Shyness: *χ*^2^ = 3.60, *df* = 1, *p* = 0.06; TI: *χ*^2^ = 0.96, *df* = 1, *p* = 0.33). There was no difference in learning speed dependent on rewarded stimuli (Mann–Whitney *U* test: *W* = 150, *p* = 0.85).

##### Reversal learning

Differences in learning speed among adult females in the reversal learning task (range, 24–100; mean ± SE, 65.22 ± 4.53) were explained by exploration (Exploration: *χ*^2^ = 7.00, *df* = 1, *p* = 0.008, estimate = 9.85, Fig. 1b), though here the association between the two traits was reversed compared to chicks: less explorative female fowl were faster at reversal learning (Fig. 1b). Similar to chicks, neither shyness nor tonic immobility predicted learning speed in the reversal learning task (Shyness: *χ*^2^ = 1.86, *df* = 1, *p* = 0.17; TI: *χ*^2^ = 0.25, *df* = 1, *p* = 0.61). Adult hens learned to associate both stimuli with a reward equally fast (Mann–Whitney *U* test: *W* = 0.94, *p* = 0.13).

## Discussion

In the current study, we investigated the relationship between learning speed and personality in male and female chicks, and adult female red junglefowl, by using multiple cognitive tasks and personality assays. We observed large differences between individuals in learning speed in all cognitive tasks, but no rank order consistency in learning speed within individuals across different learning tasks neither in chicks nor in adults. Female chicks learned faster than males in a reversal learning task, but no sex differences were apparent in discrimination and learning of location in an arena. Additionally, we found a task- and age-dependent relationship between learning speed and personality; explorative chicks, as opposed to less explorative adult females, were faster at reversal learning.

It has recently been suggested that studies in cognition should focus on individual variation (Shaw and Schmelz 2017; Boogert et al. 2018) and be investigated in tasks spanning different cognitive domains (Griffin et al. 2015), an approach not yet commonly adopted. We aimed to follow this recommendation and scored individual variation in three different learning tasks: discriminative, reversal, and spatial learning. Discrimination learning underlies the ability to associate a stimulus with appetitive or aversive stimuli, reversal learning involves the capability of extinguishing a previously learned association to form a new one, while spatial learning measures the ability to locate a reward that is out of sight, in an arena. Of these three tasks, reversal learning is considered to be more cognitively challenging due to the extinction of previously learned cues and learning of new associations (Coppens et al. 2010). Here, performance was not correlated across different tasks; thus, we did not find support for individuals having a higher general learning ability (“g”), which has been observed in humans (e.g., Plomin 1999) and mice (e.g., Matzel et al. 2003; Light et al. 2011; see review by Shaw and Schmelz 2017). While this warrants further investigation to rule out in the red junglefowl, it is supported by recent work in a larger sample (˃ 300 chicks) in the same population where discriminant and reversal learning was found to be weakly, negatively correlated (Sorato et al. 2018). This suggests that there is neither general learning performance nor a strong trade-off in learning speed between different learning tasks. Moreover, previous studies have provided mixed evidence for the existence of such trade-offs (e.g., Boogert et al. 2011; Guillette et al. 2015; Shaw et al. 2015; Bebus et al. 2016; Andersson et al. 2017). This may be explained, at least in part, by the rather large differences between studies in, for example, study design, task types used, age, and sex of the subjects. On the other hand, there could also be species-specific patterns in cognitive performance in different tasks, and relationships across tasks.

We observed a spontaneous preference for blue over green in a discrimination task in chicks, while we observed no preference in the reversal learning task for chicks nor for the black and white patterns used in adults. Interestingly, a spontaneous preference for blue has previously been observed in birds (domestic chicks, *Gallus gallus domesticus*, over green, Fischer et al. 1975, and over red, New Zealand robins, Shaw et al. 2015) and resulted in faster learning in wild New Zealand robins (Shaw et al. 2015). Although the color cues used in our study did not change other relationships recorded, this suggests that using colors in avian discrimination learning can be problematic and that the choice of color should be considered carefully when planning learning experiments in avian species.

In our study, we observe no sex difference in simpler tasks, but individual differences in learning speed were to some extent explained by sex for reversal learning where male chicks needed more trials to complete the task than females. Red junglefowl are sexually dimorphic where males are larger and more ornamented than females (Zuk et al. 1990) and sex differences in behavior are often observed (e.g., Nätt et al. 2014; Favati et al. 2016; Zidar et al. 2017a). Here we showed that differences between the sexes could be detected already in very young chicks (3–6 days post-hatching, but see Sorato et al. 2018 that in a larger sample did not find this). In domestic fowl, females seem more behaviorally flexible, while males are more persistent, which have been suggested to be linked to effects of testosterone on cholinergic activity in brain regions that are implicated in memory (Rogers 1974). When a response becomes inappropriate in a task, it might therefore be easier for females to change their response, compared to males. Sex differences in behavioral flexibility were however not detected in young chicks of our population (Zidar et al. 2017a), although we have not yet explored their physiological differences. Sex-specific learning speed is observed in a range of species tested across a range of learning tasks and setups (e.g., Range et al. 2006; Titulaer et al. 2012; Brust et al. 2013; Carazo et al. 2014), where males usually need fewer trials than females to learn a task (but see Brodin and Urhan 2015). Differences in study design and the type of tasks that are employed may explain to some extent these discrepancies. Furthermore, sex differences may vary across species depending on, for example, ecology and social organization generating different theoretical predictions on sex differences in aspects of cognition. Future studies should explore species-specific differences in sex-specific patterns of learning speed, which may be related to variation in the task used, or selection pressures the sexes are exposed to.

Independent of the slight sex difference in reversal learning, we observed a link between learning and personality. Speed–accuracy trade-offs are invoked to explain associations between personality and learning (Sih and Del Giudice 2012). According to this hypothesis, individuals may employ different cognitive styles (being either fast or accurate) reflecting their personality type (e.g., speed of exploration), with fast-exploring individuals being better at quickly acquiring simple learning rules and slow ones showing superior performance in processing more complex and detailed information. In agreement with this, we observed that adult female fowl that were slow explorers in a novel environment were better at learning a complex task (reversal test). However, contrary to predictions of the speed–accuracy trade-off hypothesis, we found the opposite pattern in chicks: more explorative individuals and not less explorative ones were better at reversal learning. It is possible that explorative chicks are less distracted by that they are alone in a test arena than less explorative ones. It has, for example, been shown that stressed chicks can have higher motivation for social reinstatement (Zidar et al. 2018) and that longer latencies in tonic immobility positively correlate with motivation for social reinstatement (Marin et al. 2001). In this scenario, slow-exploring chicks, which according to coping style theory are also easily stressed and fearful (Koolhaas et al. 2010), may actually be inherently better learners, as in adults, but their learning ability might be masked by motivational differences. However, in our study, we tried to reduce neophobia and anxiety in our learning tasks as much as possible by first habituating individuals to being in the arena and also to being separated from their pen mates. Moreover, chicks received as much time as they needed to make their choice, thus removing any differences in approach latencies. We therefore do not expect that neophobia has a large impact on our results. In addition, we found no association between personality traits and discriminant learning, either in chicks or adults. Thus, our results only partially support a speed–accuracy trade-off, suggesting that the relationships between learning speed and personality may be more complexly interlinked.

Studies investigating a link between cognition and personality have focused mainly on correlating performance in a single cognitive task to a single score obtained in a personality assay. Several of these confirmed a relationship between learning and personality (e.g., mice, Morris 1984; guppies, Dugatkin and Alfieri 2003; black-capped chickadees, Guillette et al. 2009, 2011, 2015; great tits, Exnerová et al. 2010; Amy et al. 2012; Titulaer et al. 2012; zebra finches, Brust et al. 2013; Florida scrub-jays, Bebus et al. 2016), and similar to our findings, the relationship is often limited to more cognitively demanding tasks (i.e., reversal learning: Guillette et al. 2011; Titulaer et al. 2012; Brust et al. 2013; but see Dogherty and Guilliette 2018). The reason why cognitively demanding tasks are more often associated with personality than simpler tasks is not clear. Higher demands on attention have been suggested as one possible aspect of complex tasks that can explain personality differences (Titulaer et al. 2012) and should be investigated further.

We observed a shifting relationship between reversal learning and personality over time, which to our knowledge has not previously been documented. It is important to consider that the learning tasks we conducted on chicks and adults had methodological differences. For example, different colored cues were used for chicks and adults, and when we tested adult females, they were trained also after they reached our set learning criterion, while chicks only trained until they reached our learning criterion. Thus, even though tasks were designed to capture the same aspect of learning, we cannot exclude that differences between task implementations in chicks and adults may have influenced the outcome and potentially explain the opposite pattern in chicks and adults see Cauchoix et al. 2018. If the differences in methodology explain the observed shift in the relationship between personality and cognition, it raises important implications for research in the field. Most studies differ slightly in task design both within and across species, which may hamper comparison across studies. On the other hand, we observe particularly exploration and reversal learning to be related, in both chicks and adult female. In the red junglefowl, shifting relationships between cognition and personality over ontogeny would not be unexpected. Red junglefowl go through several stages in ontogeny (e.g., from chicks are dependent on their mother to increased independence, sexually immature to sexually mature, see references in Favati et al. 2016) that have the potential to independently affect personality (Favati et al. 2016), and learning speed within tasks was here not strongly correlated over time (but see Cauchoix et al. 2018). Further, early experiences can have long-lasting effects and affect personality traits later in life (Zidar et al. 2017b), suggesting a potential ongoing interplay between personality and cognition. However, the explanation to the observed shift in the association between personality and learning speed is currently unclear and begs for further empirical and theoretical investigation.

## Conclusion

We did not observe learning speed across tasks to inter-relate. In reversal learning, male chicks were somewhat slower learners than female chicks. We observed a relationship between cognition and personality, which is task- and age-dependent: learning speed in reversal learning and exploration of a novel arena related in both chicks and adults where explorative chicks and slower exploring females learned faster. Our results thus only partly support a speed–accuracy trade-off suggesting that also other explanations may underlie observed variation in learning. Taken together, our work adds to the growing literature on individual variation in personality and cognition, and the fowl as a model species for this research (Garnham and Løvlie 2018).

## Electronic supplementary material

ESM 1 (PDF 737 kb)

Supplementary Table 2 (XLSX 39.1 kb)
