# Supplementary material for: The relationship between learning speed and personality is age- and task-dependent in red junglefowl
Source: Behav Ecol Sociobiol. 2018 Sep 26;72(10):168. doi: 10.1007/s00265-018-2579-2 (PMC6182743; doi:10.1007/s00265-018-2579-2)
Supplement: Supplementary file 1 — (PDF 737 kb) [file 265_2018_2579_MOESM1_ESM.pdf]

Supplementary material

The relationship between learning speed and personality is age- and task-dependent in red junglefowl

Josefina Zidar[a], Alexandra Balogh[a], Anna Favati[b], Per Jensen[a], Olof Leimar[b], Enrico Sorato[a], and Hanne Løvlie[a*]

[a]IFM Biology, Linköping University, SE-581 83 Linköping, Sweden

[b]Department of Zoology, Stockholm University, SE-106 91 Stockholm, Sweden

*Corresponding author, telephone number: 0046-13 281332, fax number: 0046-13281399, email: hanne.lovlie@liu.se

Behavioral Ecology and Sociobiology

The supplementary material contains:

1) Description and analyses of a side preference test

2) Supplementary figures

2a) Figures related to learning tasks

- Figure S1. Schematic of the arena used for chicks in associative and reversal learning tasks.
- Figure S2. Box-plot showing observed differences in side preference bias in chicks.
- Figure S3. Schematic of the arena used in a spatial learning task for chicks.
- Calculation of learning curves.
- Figure S4. Learning curves for female fowl in associative- and reversal learning tasks.
- Figure S5. Scatterplots of learning speed in cognitive tasks

2b) Figures related to personality assays

- Figure S6. Schematic of the arena used in the novel arena- and novel object tests for chicks and adults.
- Figure S7. Principal component biplot with individual chicks and variables displayed.
- Figure S8. Principal component biplot with individual adult females and variables displayed.

3) Supplementary tables

- Table S1. Rank order consistency in behavioural responses from repeated personality assays.
- Table S2. Raw data collected and used for analyses. Please see separate file, submitted online to Behavioral Ecology and Sociobiology’s home page.

1) Description and analyses of a side preference test

Method

To investigate if the red junglefowl chicks had a strong side-preference bias (Rodgers 1995), chicks were singly tested at day 6 post-hatching in the same arena as used for the associative and reversal learning task. Both stimuli had the same colour and both sides were rewarded. The colour used was the colour the chick had most recently associated with a reward (i.e. the colour rewarded for in the reversal learning task, except for the four chicks that did not learn that task, where the colour they were trained on during the associative learning task was used instead). The 10 subsequent side-choices a chick made were recorded and the ratio of right vs. left was used as a measure of side preference bias. The test was repeated again when the chicks were 17 days old, which is post-lateralization (see Rodgers 1995 for more detailed information on lateralization of the chicken brain) of the brain, and prior to the spatial learning task.

Statistical analysis

To investigate whether chicks had a preference for either the right or the left side, pre- and post-lateralization, we analysed individual side preference, using Mann-Whitney U tests. Additionally, we examined if any preference for right or left side had changed post-lateralization by comparing the choices the chick made in the first (pre-) and second side-preference test (post-lateralization), using a Chi-square test.

Results

Chicks showed no preference for the left or right-side pre-lateralization ($W = 2345.50$, $p = 0.15$). However, post-lateralization, there was a preference for the right side ($W = 1503.00$, $p = 0.009$). There was a tendency of a shift from left to right between pre- and post-lateralization ($\chi^2 = 5.43$, df = 2, $p = 0.07$, see figure S2).

Reference

Rogers LJ. 1995 *The development of brain and behaviour in the chicken.* Wallingford, UK: CAB International.

2) Supplementary figures

2a) Figures related to learning tasks

Figure S1. Schematic of the arena (18×37×28 cm.) used in a discriminative and reversal learning task for red junglefowl chicks. At the far end of the arena two coloured bowls were presented with coloured signs behind them. The signs were used to make sure that the chicks saw the colours from the starting position, even when being small. The coloured bowls were separated by a small cardboard partition (ca 5 cm.) so that the chick had to make a distinct choice between the colours (i.e. a chick would not be able to see where the mealworm was before making its choice). This way the chick had to walk from the starting position (marked with an x) up to the chosen bowl to make its decision.

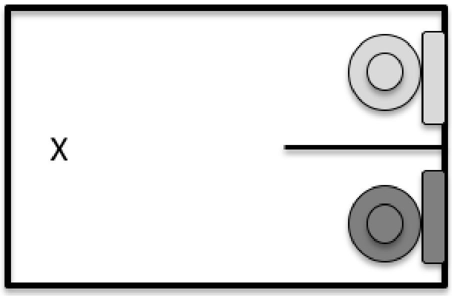

Figure S2. Side preference observed in red junglefowl chicks in the side preference test, showing proportion of choices in a preferences bias test a) before and b) after lateralization of the brain. Significance are shown with asterisks *p < 0.05 level, ** p < 0.01, *** p < 0.001.

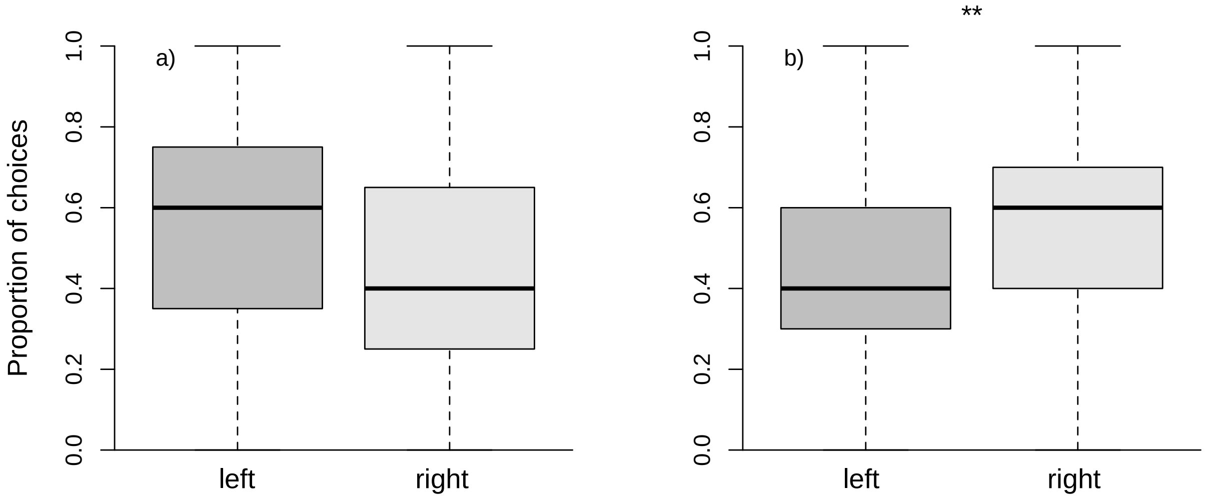

Figure S3. Schematic of the arena (76×114 cm) used in a spatial learning task for red junglefowl chicks. The starting position is marked with an x. A circle represents the placement of a bowl containing a reward. The black line represents a solid wall, and the thick patterned line represents the metal grid. A dashed line shows the correct pathway to the reward.

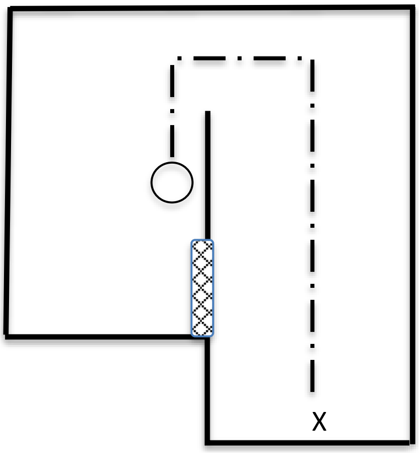

Calculation of learning curves

To confirm that adult females learned discriminative and reversal learning tasks we exposed them to, we constructed learning curves. Because the birds were presented with two colours at the same time their response can be analysed as a bivariate response (correct, incorrect). To visualise learning over time we analysed learning in blocks of 5 choices. For discriminative learning we included data from learning step 3, i.e. when birds flipped the lid on their own and could not see the reward (see main manuscript for more details). We fitted generalized linear models with a binomial distribution and logit function, using number of correct choices over total number of choices as response variable and block (i.e. 1-5, 6-10, 11-15..) as predictor.

Figure S4. Learning curve of adult red junglefowl females for a) discriminant learning (discarding step 1 and 2 in the learning process, see main text for details), b) reversal learning. In both cases individuals show increased proportion of correct choices during testing, indicating learning.

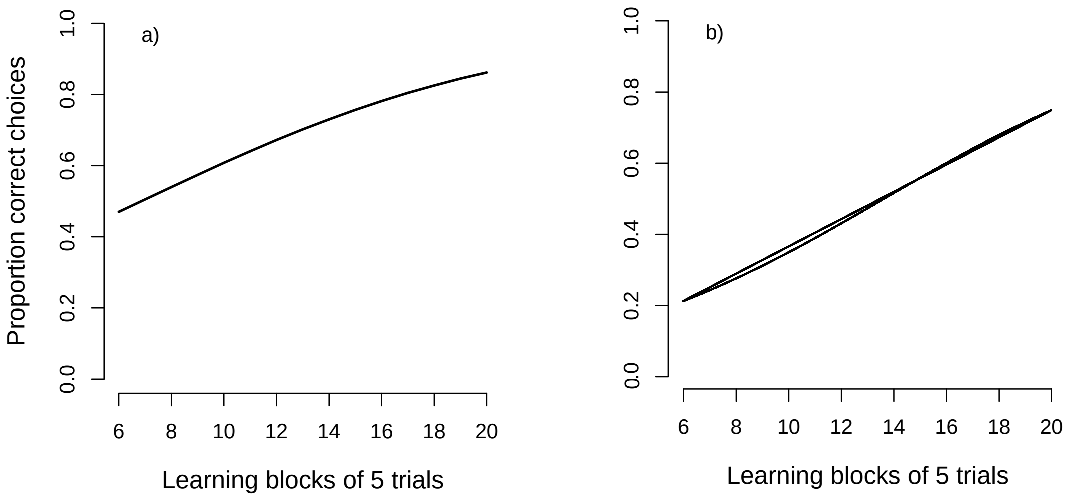

Figure S5. Scatterplots showing learning speed within and across cognitive tasks for chick and adult red junglefow. Within tasks for a) discriminative learning, and b) reversal learning. Across tasks for c) discriminate and reversal learning in chicks, d) discriminative and spatial learning in chicks and e) reversal and spatial learning in chicks, as well as for f) discriminate and reversal learning in adult red junglefowl. Females = black circles, males = grey circles.

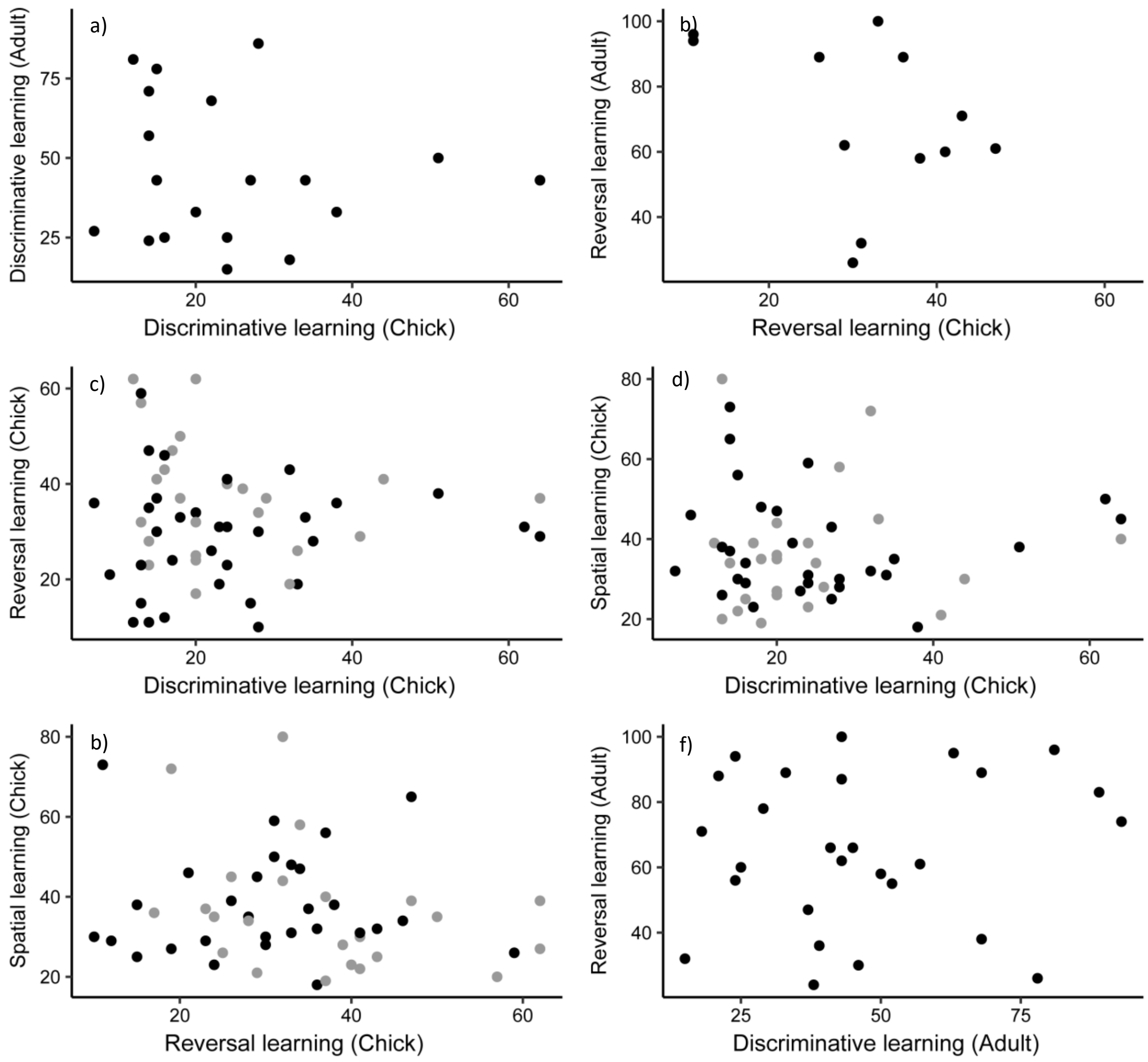

2b) Figures related to personality assays

Figure S6. Schematic of a a) first, and b) second novel arena (76×114 cm) used to score personality of red junglefowl chicks, and c) third novel arena (200×200 cm) used to score personality of adult red junglefowl. Circles represents placement of familiar food and water containers.

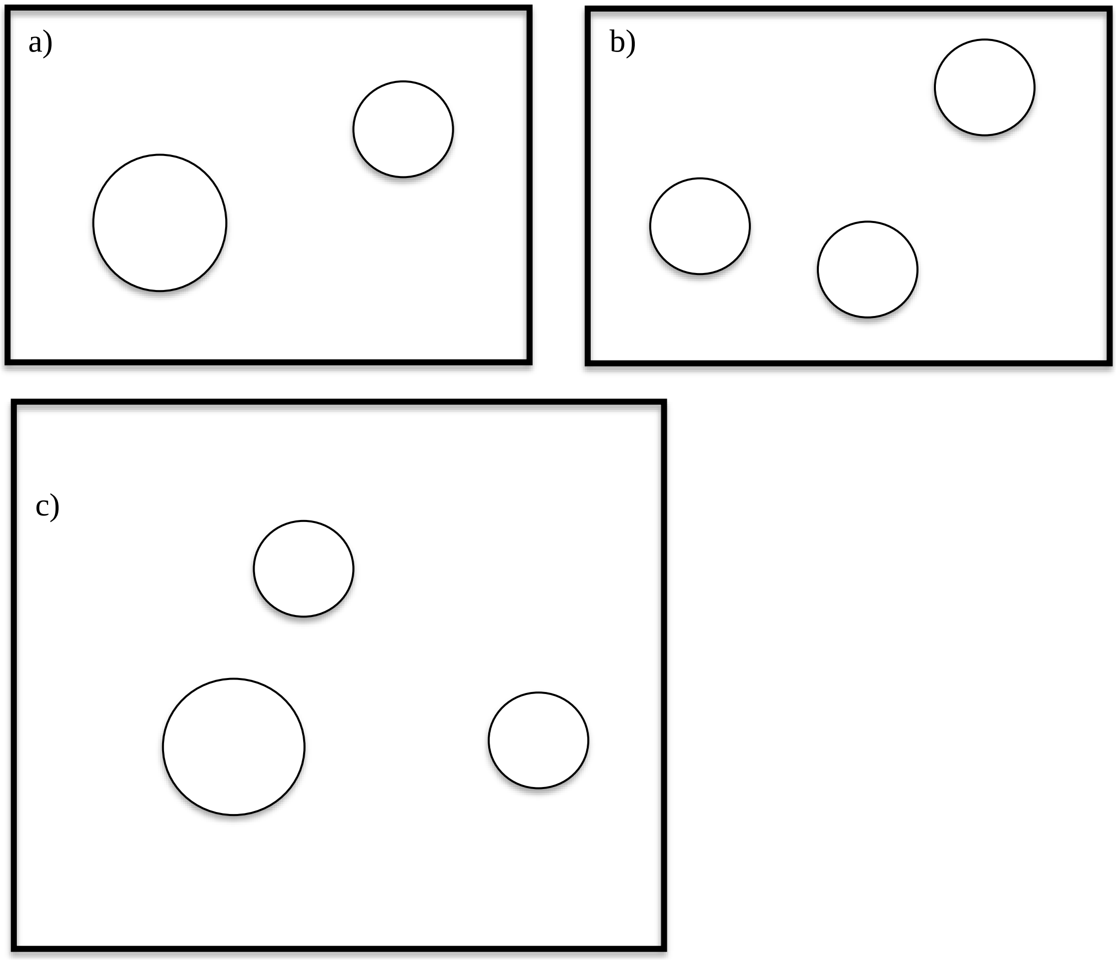

Figure S7. Principal component biplot with individual red junglefowl chicks and variables displayed. Purple triangles represent individual males and green circles individual females. The variance explained by the component is presented in brackets on the axis for that component. PC1 was interpreted as describing variation in exploration and PC2 as describing variation in shyness. NA = novel arena, NO = Novel object, Lat_Move = Latency until a chick started moving after being introduced in the arena, Lat_Explore = latency until a chick had explored the entire arena, Locomotion = proportion of time walking and running in the arena Vig = proportion of time being vigilant (i.e. standing with eyes open and head above shoulder-height), Escape = number of escape attempts.

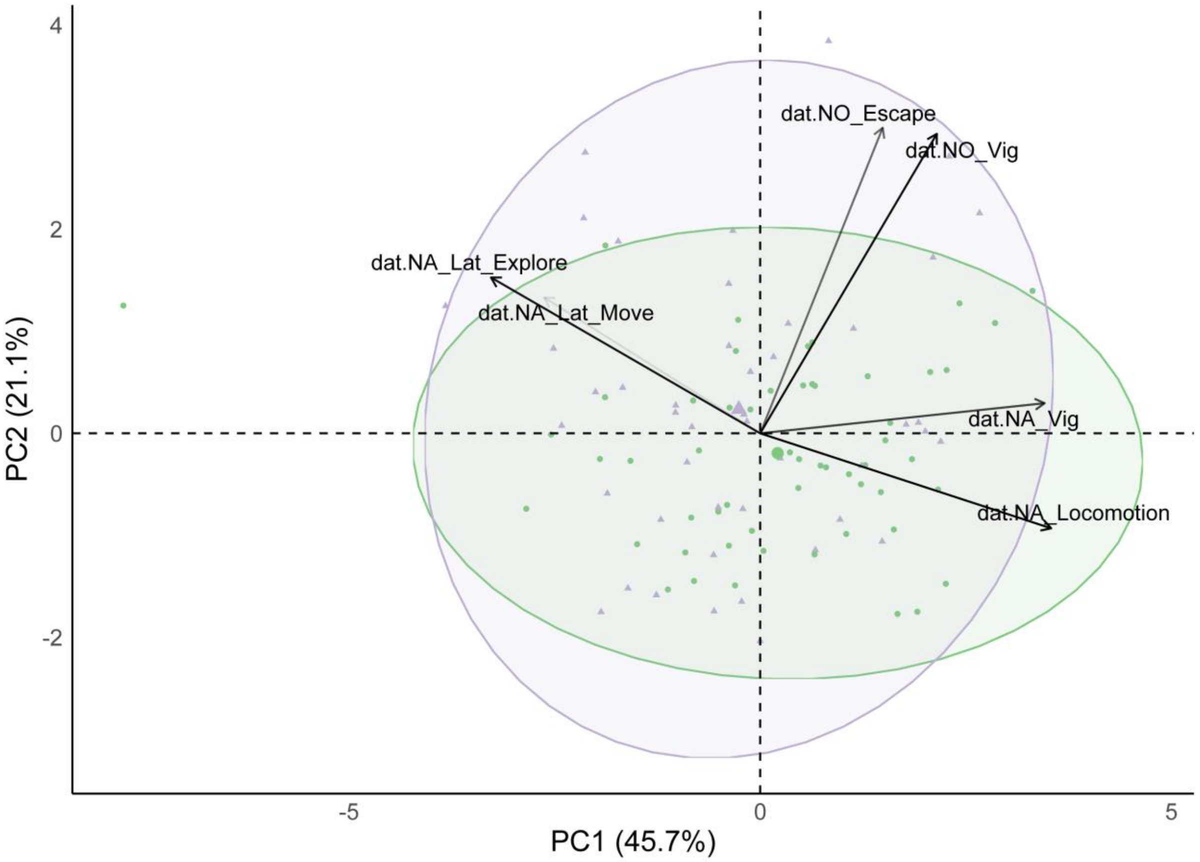

Figure S8. Principal component biplot with individual adult red junglefowl females and variables displayed. Purple triangles represent individual males and green circles individual females. The variance explained by the component is presented in brackets on the axis for that component. PC1 was interpreted as describing variation in exploration and PC2 as describing variation in shyness. NA = novel arena, NO = Novel object, Lat_Move = Latency until a chick started moving after being introduced in the arena, Lat_Explore = latency until a chick had explored the entire arena, Locomotion = proportion of time walking and running in the arena Vig = proportion of time being vigilant, i.e. standing with eyes open and head above shoulder-height, Escape = number of escape attempts.

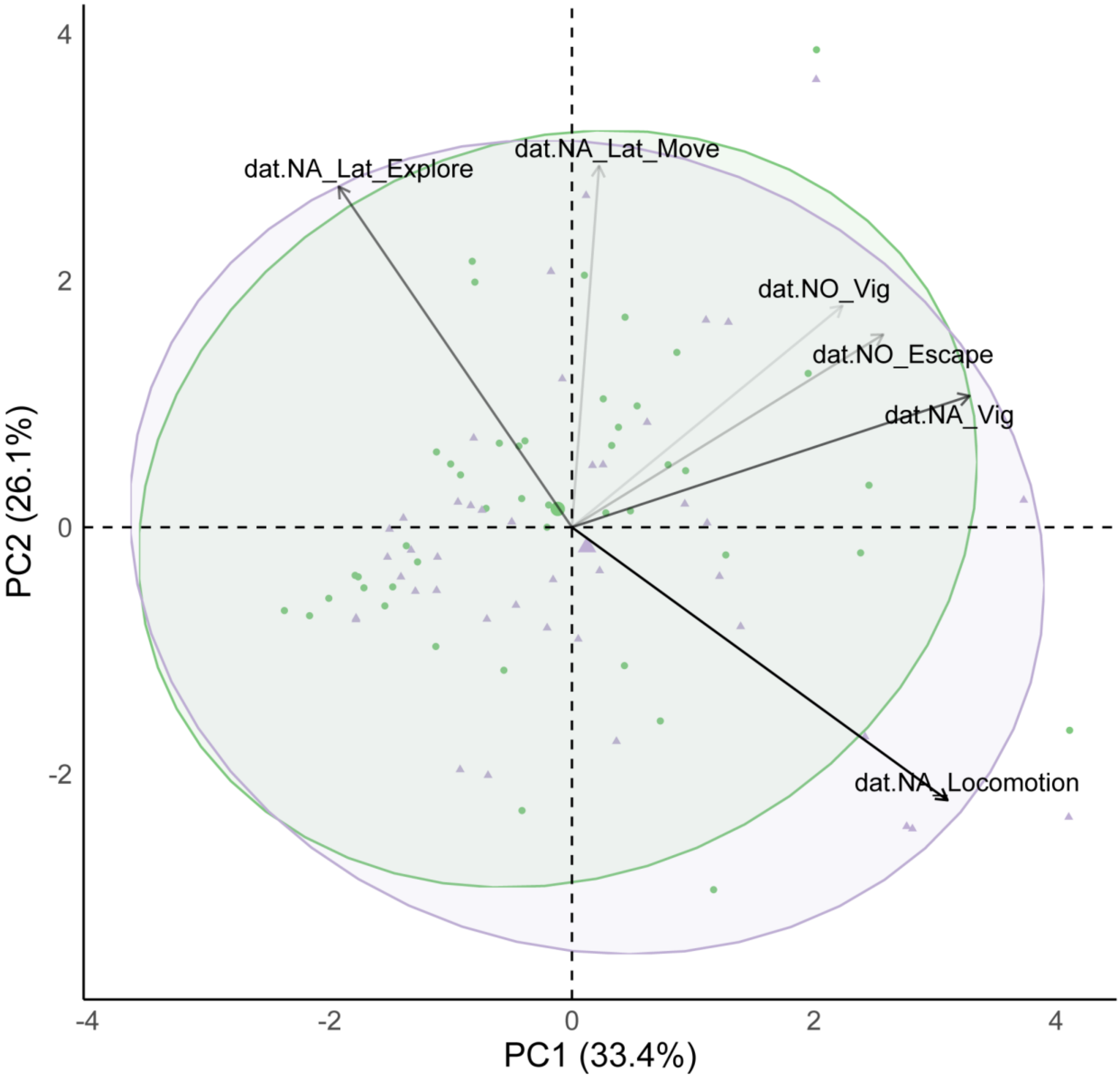

Supplementary table

Table S1. Rank order consistency in behavioural responses of red junglefowl in repeated personality assays (Novel arena, NA, Novel object, NO and Tonic immobility, TI).

Age	4 weeks-6 weeks	4 weeks-Adult	6 weeks-Adult
Locomotion	0.49** / 0.45* / 0.46***	-0.02 / 0.12 / 0.04	0.04 / -0.09 / -0.1
Vigilance (NA)	0.13 / 0.21 / 0.20	0.04 / 0.13 / 0.09	0.07 / 0.09 / 0.07
Latency to move (NA)	0.30 / 0.21 / 0.26*	-0.01 / 0.06 / 0.01	0.09 / 0.26 / 0.14
Latency to explore (NA)	0.27 / 0.29 / 0.28*	0.23 / 0.13 / 0.18	0.16 / 0.02 / 0.06
Vigilance (NO)	0.55*** / 0.23 / 0.39***	0.14 / 0.27 / 0.21	0.25 / 0.22 / 0.25
Escape (NO)	0.60*** / 0.37 / 0.48***	0.02 / -0.06 / 0.02	0.36 / 0.22 / 0.29*
Latency to move (TI)	-0.06 / 0.34 / 0.21	0.09 / -0.14 / -0.03	-0.01 / 0.21 / 0.14

Spearman rank correlations coefficients are given for males/females/both sexes combined. Asterisk signifies significance at the *$p < 0.05$ level, ** $p < 0.01$, *** $p < 0.001$ after correction for multiple testing. $n_{chicks(male/female)}$ = 45/55, $n_{adults(male/female)}$ = 42/45.
